# Clinical features and prognosis of acute-on-chronic liver failure in patients with recompensated cirrhosis

**DOI:** 10.1186/s12876-023-02956-4

**Published:** 2023-09-19

**Authors:** Haixia Yuan, Yingying Cao, Zhenjun Yu, Yue Huang, Fang Liu, Yanying Gao, Shaotian Qiu, Tao Han

**Affiliations:** 1 Department of Hepatology and Gastroenterology, The Third Central Clinical College of Tianjin Medical University, Tianjin, 300170 China; 2 Department of Hepatology and Gastroenterology, Tianjin Union Medical Center affiliated to Nankai University, 190 Jieyuan Road, Hongqiao District, Tianjin, 300121 China; 3 Department of Hepatology and Gastroenterology, The Third Central Hospital of Tianjin, 83 Jintang Road, Hedong District, Tianjin, 300170 China

**Keywords:** Acute-on-chronic liver failure, Liver cirrhosis, Recompensation, Clinical feature, Prognosis

## Abstract

**Background:**

There are few studies on acute-on-chronic liver failure (ACLF) in patients with recompensated cirrhosis. This study was aimed to investigate the clinical features of ACLF patients with recompensated cirrhosis.

**Methods:**

A total of 461 ACLF patients were enrolled and divided into three groups: compensated, recompensated, and decompensated cirrhosis with ACLF. The baseline clinical data and 1-year survival rates were compared among the three groups.

**Results:**

Compared with the decompensated group, in the recompensated group, the levels of hemoglobin, albumin, and serum sodium were significantly higher and the white blood cell count, international normalized ratio, and incidence of respiratory failure were significantly lower; there were no evident differences in other organ failures. The proportion of patients with ACLF grade 3 and 1-year survival rates significantly differed between the two groups. Conversely, compared with the compensated group, in the recompensated group, the platelet and total bilirubin levels were significantly lower and the proportion of patients with ACLF grade 1 was significantly higher. However, other clinical indicators or 1-year survival rates did not significantly differ between the two groups.

**Conclusions:**

Compared with patients who developed ACLF with decompensated cirrhosis, those who developed ACLF with recompensated cirrhosis had a less severe condition, lower incidence of respiratory failure, and better 1-year prognosis. However, the baseline clinical features and prognosis were similar between ACLF patients with recompensated and compensated cirrhosis.

**Trial registration:**

Chinese clinical trials registry: ChiCTR1900021539.

**Supplementary Information:**

The online version contains supplementary material available at 10.1186/s12876-023-02956-4.

## Introduction

Liver cirrhosis is characterized by diffuse fibrosis, pseudolobular formation, and vascular proliferation and is classified into compensated and decompensated stages. In recent years, many researchers have realized that liver cirrhosis is a dynamic process with multiple clinical manifestations and different prognoses and requires further detailed staging [1]. Therefore, hepatologists from China believe that liver cirrhosis can be divided into not two but three stages, namely compensated, decompensated, and recompensated stages. The recompensation phase is defined as a condition wherein due to the effective control of etiology as well as treatment or prevention of complications, decompensated cirrhosis patients can no longer experience decompensation events for a long time (at least 1 year). However, they may still have the clinical and laboratory characteristics of compensated liver cirrhosis, which is regarded as “recompensation” [2].

Acute-on-chronic liver failure (ACLF) is a syndrome with acute decompensation accompanied by organ failure and a high short-term mortality rate associated with chronic liver disease. In clinical practice, patients with recompensated cirrhosis and compensated cirrhosis were found to have similar clinical characteristics [3]. The concept of recompensation is relatively new, and in its absence in the past, it was impossible to distinguish the clinical features and prognosis of ACLF between patients with decompensated cirrhosis and what is now known as recompensated cirrhosis. Therefore, we know little about the clinical features and prognosis of ACLF patients with recompensated cirrhosis.

This study aimed to investigate the clinical features and prognosis of ACLF patients with recompensated cirrhosis by comparing them with ACLF patients with compensated and decompensated cirrhosis.

## Patients and methods

### Study participants and data collection

**Fig. 1:**
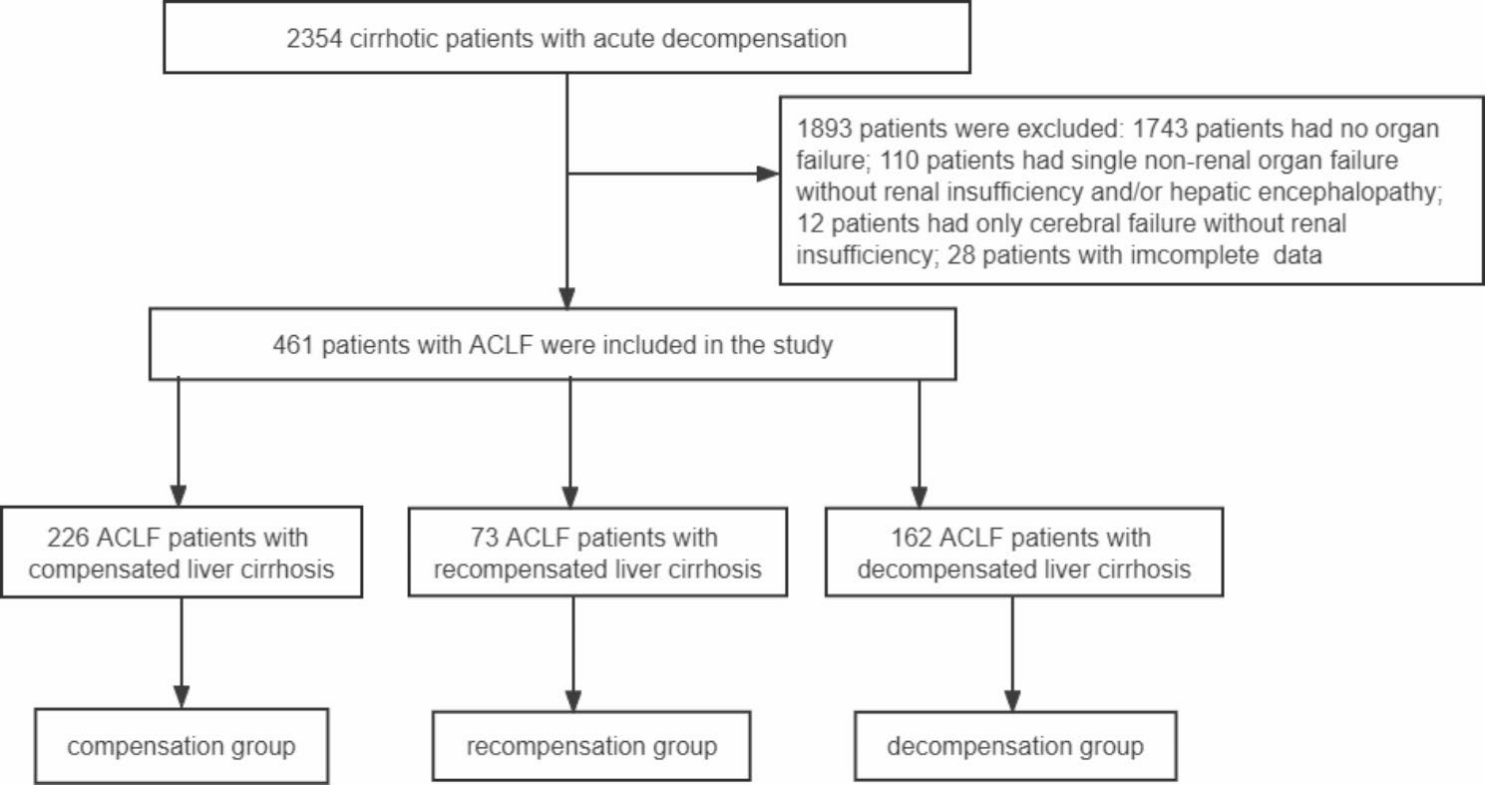
Flow chart of ACLF patient(s) enrollment. ACLF, acute-on-chronic liver failure

The data of patients with acute decompensated liver cirrhosis admitted to Tianjin Third Central Hospital between May 2009 and May 2019 were retrospectively collected, and 461 patients who met the definition of ACLF as per the European Association for the Study of the Liver-chronic liver failure (EASL-CLIF) consortium were selected as the research participants (Fig. 1).

Compensated cirrhosis is defined as liver biopsy findings indicating a diagnosis of liver cirrhosis or endoscopic examination showing esophagogastric varices or ectopic varices of the digestive tract (except non-cirrhotic portal hypertension), or imaging features suggest cirrhosis or portal hypertension [2]. Decompensated cirrhosis is characterized by portal hypertension and/or hepatic dysfunction, which is accompanied by ascites, portal hypertensive hemorrhage, and hepatic encephalopathy, among other complications. Recompensated cirrhosis is defined as a condition wherein due to the effective control of etiology and treatment or prevention of complications, decompensated cirrhosis patients can no longer experience decompensation events for a long time (at least 1 year), thus reversing to “recompensation” [2].

Based on the definition of ACLF developed by the EASL-CLIF consortium, patients were divided into three groups: ACLF with compensated cirrhosis, recompensated cirrhosis, or decompensated cirrhosis.

According to the ACLF grade of the EASL-CLIF consortium, the patients were divided into three grades [4].

ACLF grade 1: single kidney failure or single non-kidney organ failure (liver, coagulation, circulation, or lungs) with kidney insufficiency (serum creatinine level: 1.5–1.9 mg/dL) and/or mild to moderate hepatic encephalopathy (grade I or II according to the West Haven classification); single cerebral failure with serum creatinine level ranging from 1.5 to 1.9 mg/dL. ACLF grade 2: two organ failures. ACLF grade 3: three or more organ failures. The exclusion criteria were the presence of severe extrahepatic chronic diseases, concomitant human immunodeficiency virus infection, liver cancer or other malignancies, pregnancy, or history of liver transplant.

The general information (e.g., age, sex, and etiology of liver cirrhosis) and findings of routine blood tests, liver function tests, kidney function tests, blood coagulation function tests, serum sodium levels, and arterial blood gas analysis of the patients were collected. Examination data obtained during hospitalization, such as liver biopsy, ultrasound, CT, MRI, and gastroscopy, were also recorded. All patients were followed up for 1 year after the date of ACLF diagnosis, and the outcomes included survival and death. This study was approved by the Ethics Committee of Tianjin Third Central Hospital and conducted in compliance with the principles of the Declaration of Helsinki [ethics committee approval no. SZX-IRB-SOP-016(F)-002-01].

### Statistical analysis

Continuous data were reported as $\stackrel{-}{x}\pm s$ or $M({P}_{25 }\sim{P}_{75})$. The F test or K-W test was used for comparisons among the three groups, and the Bonferroni test was used for pairwise comparisons. Categorical variables were reported as frequency (%). The Chi-squared test or Fisher’s exact test was used for comparisons among three groups, and the Bonferroni test was used for pairwise comparisons. Kaplan–Meier survival curves were used to describe the survival distributions, and the Log-rank test was used to compare the differences in survival curves. Two-tailed *P* values <0.05 were considered statistically significant for all tests. Statistical analyses were performed using SPSS Statistics 23.0 (IBM Corp., Armonk, New York, USA).

## Results

### Clinical characteristics of patients

We herein studied 461 ACLF patients who met the EASL-CLIF consortium criteria, including 376 men (81.6%). The overall average age of patients was 52.1 ± 10.9 years. The etiologies of cirrhosis were hepatitis B-related cirrhosis in 227 cases (49.2%), alcoholic cirrhosis in 184 cases (39.9%), and other causes of cirrhosis (e.g., hepatitis C cirrhosis and cholestatic cirrhosis) in 50 cases (10.9%). The numbers of ACLF patients in the three groups were as follows: 226 cases (49.0%) in the ACLF with compensated cirrhosis group, 73 cases (15.8%) in the ACLF with recompensated cirrhosis group, and 162 cases (35.1%) in the ACLF with decompensated cirrhosis group. The proportion of patients with other causes of cirrhosis was higher in the recompensated group than in decompensated and compensated groups (20.5% vs. 8.6% vs. 9.3%, in that order; *P* = 0.014), whereas that of hepatitis B cirrhosis was lower in the recompensated group than in the compensated group (38.4% vs. 54.4%, *P =* 0.044). There were significant differences in the precipitating events of ACLF among the three groups. The proportion of ACLF patients precipitated by bacterial infection was higher in the decompensated group than in recompensated and compensated groups (Table S1).

### Comparison of baseline clinical laboratory indicators among the three groups of patients

There were significant differences in the following parameters among the three groups: international normalized ratio (INR), white blood cell (WBC) counts, and levels of hemoglobin (Hb), platelet count (PLT), total protein (TP), albumin (ALB), total bilirubin (Tbil), alanine aminotransferase (ALT), aspartate aminotransferase (AST), alkaline phosphatase (ALP), cholinesterase (CHE), creatinine (Cr), and serum sodium. Hb, ALB, and serum sodium levels were significantly higher in the recompensated group than in the decompensated group, whereas WBC and INR were significantly lower in the former than in the latter. Compared with the compensated group, PLT and Tbil were significantly lower in the recompensated group; however, there were no significant differences in WBC, Hb, ALB, ALT, AST, ALP, CHE, INR, Cr, and serum sodium between the two groups (Table S1). In this study, to exclude the effect of esophagogastric variceal bleeding (EVB) on Hb, ACLF patients with compensated cirrhosis, decompensated cirrhosis, and recompensated cirrhosis precipitated by EVB were compared, and no significant difference in Hb was found among the three groups (Table S2).

### Comparison of organ failure and ACLF grade among the three groups of patients

In terms of organ failure, the incidence of liver, circulatory, and respiratory failure significantly differed among the three groups (*P* < 0.05). Compared with the decompensated group, the proportion of respiratory failure was significantly lower in the recompensated group (5.5% vs. 22.8%, *P* < 0.001); however, the failure rates of other organs did not significantly differ between the two groups. Compared with the compensated group, in the recompensated group, the occurrence rate of circulatory failure was higher (13.7% vs. 4.9%, *P* = 0.01) and that of liver and respiratory failure were lower (53.4% vs. 84.1%, *P* < 0.001; 5.5% vs. 16.8%, *P* = 0.015). The ACLF grade significantly differed among the three groups (*P* < 0.05). The proportion of patients with ACLF grade 3 was lower in the recompensated group than in the decompensated group (15.1% vs. 35.8%, *P* < 0.001), and the proportion of patients with ACLF grades 1 and 2 did not significantly differ between the two groups. The proportion of patients with ACLF grade 1 was significantly higher in the recompensated group than in the compensated group (41.1% vs. 20.8%, *P* < 0.001), and there were no significant differences in the proportion of patients with ACLF grades 2 and 3 between the two groups (Table 1).

**Table 1:** Comparison of organ failure and ACLF grade in the three groups of patients

Characteristic	Compensated group (n = 226)	Recompensated group (n = 73)	Decompensated group (n = 162)	*χ* ^*2*^	*P*
*Organ failures*					
Liver (n, %)	190 (84.1%)	39 (53.4%)	81 (50.0%)	57.252	< 0.001
Kidney (n, %)	65 (28.8%)	17 (23.3%)	57 (35.2%)	3.789	0.150
Cerebral (n, %)	55 (24.3%)	13 (17.8%)	31 (19.1%)	2.205	0.332
Coagulation (n, %)	141 (62.4%)	45 (61.6%)	118 (72.8%)	5.302	0.071
Circulation (n, %)	11 (4.9%)	10 (13.7%)	37 (22.8%)	27.810	< 0.001
Lungs (n, %)	38 (16.8%)	4 (5.5%)	37 (22.8%)	10.713	0.005
*ACLF grade*					
Grade 1 (n, %)	47 (20.8%)	30 (41.1%)	44 (27.2%)	11.853	0.003
Grade 2 (n, %)	120 (53.1%)	32 (43.8%)	61 (37.7%)	9.249	0.010
Grade 3 (n, %)	59 (26.1%)	11 (15.1%)	58 (35.8%)	11.395	0.003

### Comparison of prognosis among the three groups

**Fig. 2:**
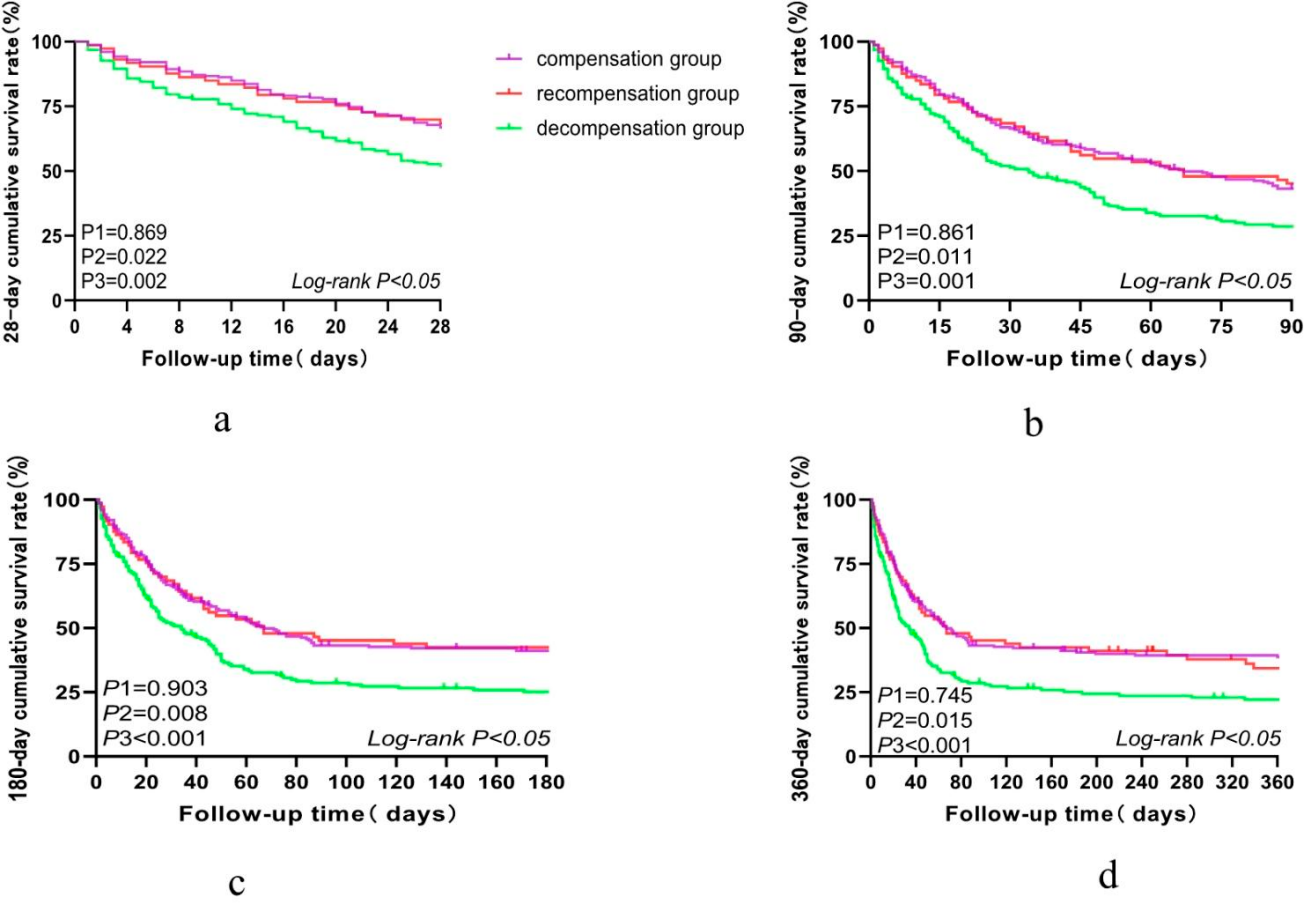
**a-d**, Comparison of 28-day, 90-day, 180-day, and 1-year cumulative survival rates among the three groups of patients.*P*1, comparison between recompensated and compensated groups; *P*2, comparison between recompensated and decompensated groups; *P*3, comparison between compensated and decompensated groups

In terms of prognosis, there were significant differences in 28-day, 90-day, 180-day, and 1-year cumulative survival rates among the three groups (*P* < 0.05). There were significant differences in 28-day, 90-day, 180-day, and 1-year cumulative survival rates between recompensated and decompensated groups (68.5% vs. 52.1%, *P* = 0.022; 45.2% vs. 52.1%, *P* = 0.011; 42.5% vs. 25.1%, *P* = 0.008; 34.4% vs. 22.1%, *P* = 0.015, respectively); conversely, there were no evident differences between the recompensated and compensated groups (68.5% vs. 66.9%, *P* = 0.869; 45.2% vs. 43.2%, *P* = 0.861; 42.5% vs. 41.1%, *P* = 0.903; 34.4% vs. 38.8%, *P* = 0.745, respectively). Among the three groups, the decompensated group had the lowest cumulative survival rates at 28 days, 90 days, 180 days and 1 year (Fig. 2a, b, c and d).

**Fig. 3:**
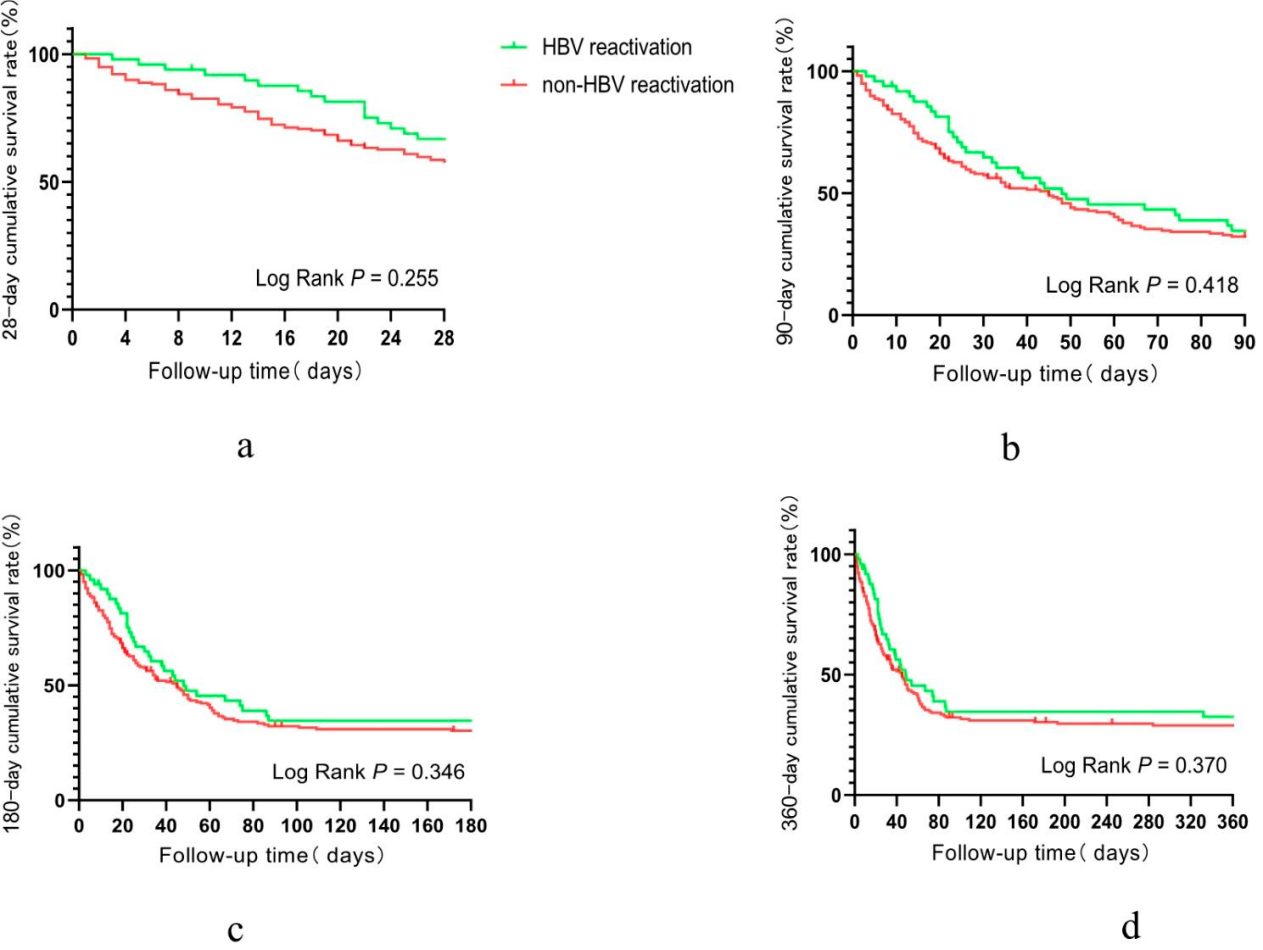
**a-d**, Comparison of 28-day, 90-day, 180-day, and 1-year cumulative survival rates of HBV-ACLF patients precipitated by hepatitis B virus reactivation and non-hepatitis B virus reactivation

In this study, there were 227 hepatitis B virus related acute-on-chronic liver failure (HBV-ACLF) patients, among whom 49 (21.6%) were precipitated by HBV reactivation. We compared the prognosis of ACLF patients precipitated by HBV reactivation and non-HBV reactivation and found no significant difference in the cumulative survival rate at 28 days, 90 days, 180 days, and a year between the two groups (Fig. 3a-d).

## Discussion

ACLF is a syndrome characterized by acute decompensation with organ failure and high short-term mortality [4–6]. Before the definition of recompensated cirrhosis was proposed, patients with recompensated and decompensated cirrhosis could not be distinguished. Therefore, we know little about the clinical characteristics and prognosis of patients having ACLF with recompensated cirrhosis. In this study, we retrospectively analyzed the medical records of 461 ACLF patients and identified that there were many features in the baseline data and prognosis of ACLF patients among those with compensated, decompensated, and recompensated cirrhosis.

Our study showed that compared with the decompensated group, the recompensated group had fewer ACLF patients precipitated by bacterial infection, and the patients had higher Hb, ALB, and serum sodium and lower WBC and INR. In the latter, the proportion of patients with respiratory failure and a diagnosis of ACLF grade 3 was significantly lower and the 28-day, 90-day, 180-day, and 1-year cumulative survival rates were significantly higher than in the former.

The CANONIC study [4] reported that patients with ACLF have a strong systemic inflammatory response, which manifests as high WBC counts and high plasma levels of C-reactive protein, and the intensity of this inflammatory response is commensurate to the severity of ACLF. The study also concluded that the WBC count was an independent predictor of ACLF-related mortality. This is consistent with the finding that WBC counts were significantly higher in the decompensated group than in the recompensated group, and the prognosis was significantly worse in the decompensated group than in the recompensated group. ACLF grade 3 refers to patients with three or more organ failures who are in severe health condition and particularly vulnerable to secondary infections, EVB, hepatic encephalopathy, and other complications. In our study, the proportions of ACLF grade 3 and respiratory failure were significantly higher in the decompensated group than in the recompensated group. Anemia is common in patients with chronic liver disease. Bihari et al. [5] discovered that systemic inflammation was associated with significantly lower levels of bone marrow hematopoietic stem cells and hemoglobin in patients with advanced liver cirrhosis. A study by Salvatore et al. [6] also supported that Hb was significantly lower in anemia patients with ACLF than in those without ACLF. Compared with patients with compensated and recompensated cirrhosis, patients with decompensated cirrhosis had a higher degree of systemic inflammation and lower Hb levels. The liver is the main organ for albumin synthesis. The ability to synthesize albumin can reportedly be reduced by 60–80% in patients with advanced liver cirrhosis [7]. In addition, albumin synthesis is also threatened by systemic inflammation and oxidative stress [8]. When decompensation or ACLF or both occur, the amount of intact albumin is significantly reduced in patients, which adversely affects their prognosis [9, 10]. Similarly, in this study, the level of ALB was significantly lower in the decompensated group than in recompensated and compensated groups, and the patients had poorer prognoses. In addition to albumin, the liver can synthesize many coagulation factors. When coagulation disorders occur in cirrhotic patients, the INR increases. Wang et al. [11] reported INR as an independent risk factor for 28-day and 90-day mortality without liver transplantation in cirrhotic patients. Therefore, compared with the recompensated and compensated groups, in the decompensated group, the INR was significantly higher and patient survival rates were significantly lower. A multicenter and prospective study reported that almost half (49.4%) of cirrhotic patients in its cohort had hyponatremia [12]. The decrease in serum sodium is correlated to the severity of liver disease. Patients with Child‒Pugh C are more likely to develop hyponatremia than those with Child‒Pugh A/B [13]. Moreover, several studies have confirmed serum sodium as an important predictor of death in patients waiting for liver transplantation [14–16]. This is consistent with the findings of the present study, wherein we showed that patients in the decompensated group had lower serum sodium and higher mortality rates than those in the recompensated and compensated groups.

In this study, compared with the compensated group, the recompensated group was associated with lower PLT and Tbil, lower rates of liver and respiratory failure, and higher rates of circulatory failure. Studies have shown that there are significant differences in the characteristics of ACLF with different etiologies. For example, alcoholic liver disease is the most common etiology of ACLF in America and European countries, and kidney failure is the most common organ failure. However, the etiology of HBV infection is more common in China, and liver and coagulation failure are the most common organ failure types [17]. The incidence of lower Tbil and liver failure was lower in the recompensated group, which may be related to the fact that the proportion of HBV-ACLF patients was significantly lower in this group than in the compensated group. The peripheral arterial vasodilation hypothesis markedly influences decompensated cirrhosis. Arterial vasodilation decreases blood pressure due to hypovolemia. Cardiovascular responses to vasoconstrictors are reduced under these circumstances [18]. Compared with the compensated group, patients in the recompensated group were less sensitive to vasoactive drugs and had a higher rate of circulatory failure. In addition, there were no significant differences in the proportion of patients with ACLF grades 2 and 3 and the cumulative survival rates of patients at 28 days, 90 days, 180 days, and 1 year between the two groups, suggesting that the clinical characteristics and prognosis of patients in the recompensated group were similar to those in the compensated group.

In this study, nearly half of the patients had HBV-ACLF, and herein, we compared the prognosis of patients precipitated by hepatitis B reactivation and non-hepatitis B reactivation and found that there was no significant difference in prognosis between the two groups within 1 year of follow-up. Consistently, a study by Yin et al. [19] also supports that although there are differences in the types of organ failure in HBV-ACLF patients with different precipitating events, they have similar short-term prognoses.

Although we compared the clinical features and prognosis of ACLF patients with different cirrhosis stages, this study also has some limitations. First, nearly half of the patients in the study had hepatitis B virus infection, and thus, the results need to be further verified in patients with different causes. Second, as this is a single-center, retrospective study, the number of patients in the recompensated group was relatively small. In the future, multicenter, prospective, and large-sample clinical studies are needed to verify the clinical characteristics and prognosis of ACLF in patients with recompensated cirrhosis.

In conclusion, although the proportion of organ failure in ACLF patients with recompensated cirrhosis was similar to that in ACLF patients with decompensated cirrhosis, the severity in the former is generally milder and the prognosis is significantly better. The baseline clinical indicators and prognosis were similar for ACLF patients with recompensated cirrhosis and ACLF patients with compensated cirrhosis.

### Electronic supplementary material

Below is the link to the electronic supplementary material.

Supplementary Material 1

Supplementary Material 2

## Data Availability

All data generated or analyzed in this study are available from the corresponding author for the reasonable request.

## Abbreviations

ACLF: Acute-on-chronic liver failure
EASL-CLIF: European Association for the Study of the Liver-chronic liver failure Consortium
WBC: White blood cell
Hb: Hemoglobin
PLT: Platelets
TP: Total protein
ALB: Albumin
Tbil: Total bilirubin
ALT: Alanine aminotransferase
AST: Aspartate aminotransferase
ALP: Alkaline phosphatase
CHE: Cholinesterase
INR: International normalized ratio
Cr: Creatinine
EVB: Esophagogastric variceal bleeding
HBV: Hepatitis B virus
