# Supplementary material for: Clinical features and prognosis of acute-on-chronic liver failure in patients with recompensated cirrhosis
Source: BMC Gastroenterol. 2023 Sep 19;23:319. doi: 10.1186/s12876-023-02956-4 (PMC10510206; doi:10.1186/s12876-023-02956-4)
Supplement: Supplementary file 1 — Supplementary Material 1 [file 12876_2023_2956_MOESM1_ESM.pdf]

Table S1 Comparison of baseline characteristics among three groups of ACLF patients

Characteristic	Compensated group（n=226）	Recompensated group（n=73）	Decompensated group（n=162）	Value	*P*
Age (years)	52. 1 ± 11.9	53.2 ± 8.9	53.9 ± 9.9	F= 1.301	0.274
Male（n,%）	182(80.5%)	60(82. 1%)	134(82.7%)	X^2=0.322	0.851
Etiology					
HBV（n,%）	123(54.4%)	28(38.4%)	76(46.9%)	X^2=6.241	0.044
Alcohol（n,%）	82(36.3%)	30(41. 1%)	72(44.4%)	X2 2=.671	0.263
Other causes （n,%）	21(9.3%)	15(20.5%)	14(8.6%)	X^2=8.484	0.014
Precipitating events					
Bacterial infection （n,%）	103（45.6%）	28（38.4%）	97（59.9%）	X^2= 11.997	0.002
esophagogastric variceal bleeding （n,%）	46（20.4%）	20（27.4%）	40（24.7%）	X^2= 1.953	0.377
HBV reactivation （n,%）	25（11. 1%）	10（13.7%）	14（8.6%）	X^2= 1.442	0.486
Other precipitating event	15（6.6%）	4（5.5%）	5（3. 1%）	X2 2=.424	0.298

(trauma/surgery/drinking,etc.)（n,%）					
No precipitating event（n,%）	67（29.6%）	15（20.5%）	22（13.6%）	X^2= 14. 142	0.001
More than one precipitating event（n,%）	30（13.3%）	4（5.5%）	17（10.5%）	X^2=3.490	0. 175
Laboratory data					
WBC（×10^9/L）	7.5(5. 1~ 11.2)	6.9(4.2~9.4)	8.5（5.5~ 12.9)	H=7.484	0.024
Hb（g/L）	111.9 ± 28. 1	109.3 ± 23.9	99.9 ± 24.2	F= 10. 104	<0.001
PLT（×10^9/L）	77.0(55.0~ 107.8)	58.0(38.0~87.0)	62.0(40.3~91.0)	H= 15. 127	0.001
TP（g/L）	89.3 ± 11.6	84.9 ± 11.0	81.4 ± 16.4	F=6.467	0.002
ALB（g/L）	28.2 ± 4.9	28.3 ± 4.5	26.6 ± 4.3	F=7.005	0.001
TBil（umol/L）	313.2(228.6~399.8)	223. 1(127.8~341. 1)	188.4(119.0~318.8)	H=56.430	<0.01
ALT（U/L）	96.5(36.0~364.0)	46.0(24.0~277.0)	43.0(24.0~ 136.8)	H= 15.771	<0.001
AST（U/L）	154.5(77.0~359.5)	89.0(52.0~296.0)	81.0(44.5~ 172.8)	H=20.299	<0.001
ALP（U/L）	132.5(99.0~ 176.3)	120.0(98.0~ 186.0)	109.5(76.0~ 153.0)	H= 10.091	0.006
CHE（U/L）	2197.5 (1559.5~3030.3)	2028.0 (1592.0~2594.0)	1852.0 (1260.5~2405.8)	H= 15.204	<0.001
INR	2.6(2. 1~3.6)	2.5(1.9~3.0)	3.0(2.4~3.9)	H= 16.383	<0.001
Cr（umol/L）	93.9(59.0~ 198.0)	105.0(65.0~ 156.0)	136.5(79.8~253.3)	H= 13.978	0.001

Na（mmol/L）	133.3(129.5~ 137.5)	133.7(130.4~ 136. 1)	131.0(126.5~ 134.6)	H= 15.916	<0.001

ALB, albumin; ALP, alkaline phosphatase; ALT, alanine aminotransferase; AST, aspartate aminotransferase; CHE, cholinesterase; Cr, creatinine; Hb, hemoglobin; INR, international normalized ratio; Na, serum sodium; PLT, platelet; TBil, total bilirubin; TP, total protein; WBC, white blood cell.
