# Supplementary material for: Clinical features and prognosis of acute-on-chronic liver failure in patients with recompensated cirrhosis
Source: BMC Gastroenterol. 2023 Sep 19;23:319. doi: 10.1186/s12876-023-02956-4 (PMC10510206; doi:10.1186/s12876-023-02956-4)
Supplement: Supplementary file 2 — Supplementary Material 2 [file 12876_2023_2956_MOESM2_ESM.pdf]

Table S2 Comparison of hemoglobin in three groups of patients with ACLF precipitated by esophagogastric variceal bleeding

Characteristic	Compensated group (n=46)	Recompensated group (n=20)	Decompensated group (n=40)	Value	*P*
Hb (g/L)	89.9±33. 1	90.3±23.2	83.5±22.8	0.692	0.503

ACLF,acute-on-chronic liver failure;Hb, hemoglobin
